# Contribution of channel geometry adjustments to stage variance based on rating-curves in the main stream of the Lancang–Mekong river

**DOI:** 10.1038/s41598-025-19822-w

**Published:** 2025-10-15

**Authors:** Li He, Qiuhong Tang, Dong Chen, Paul P. J. Gaffney, Gaohu Sun

**Affiliations:** 1 Key Laboratory of Water Cycle and Related Land Surface Processes, Institute of Geographic Sciences and Natural Resources Research, Chinese Academy of Sciences, Beijing, 100101 China; 2 University of Chinese Academy of Sciences, Beijing, 100049 China; 3 International Economic and Technical Cooperation and Exchange Center, Ministry of Water Resources, Beijing, 100053 China

**Keywords:** Channel geometry adjustment, Stage variance, Rating-curve, Hydrological regime, Lancang–Mekong river, Hydrology, Natural hazards

## Abstract

Extensive dam development and climate change have altered seasonal stage variation patterns, which are critical to residents in the riparian zone along the Lancang–Mekong River. However, the effects of morphological changes and reservoirs on stage variability are not clear due to data-scarce fluvial systems. In this work, discharge and water level data from five hydrological stations (1960–2020) were acquired to assess temporal shifts in rating curves during different disturbance periods. The contribution of channel geometry adjustment to the stage variance of the extreme flow regime under high- and low-flow conditions were empirical analysed by rating-curve method. Analysis revealed that the stage variance along the main channel was modulated by channel geometry adjustment under both high- and low-flow conditions, even though discharge was the dominant factor. Moreover, the degree of modulation resulting from geometric adjustment varied under different flow and reach conditions, which varied in the ranges of -0.57 ~ 0.27 m and − 0.41 ~ 0.39 m under low- and high-flow conditions, respectively. Furthermore, an inverse channel geometry adjustment response was observed for 60% of the high-flow conditions versus 40% of the low-flow conditions. The Luang Prabang–Vientiane reach was transitional in terms of the effects of channel geometry adjustment on stage variation. Our findings quantified how channel geometry adjustment modulated water levels across various extreme regimes, offering insights into the morphological processes of data-scarce river reaches.

**Supplementary Information:**

The online version contains supplementary material available at 10.1038/s41598-025-19822-w.

## Introduction

The Lancang–Mekong (LM) River basin has experienced increasing extremes in its hydrological properties, with intensified flood‒drought cycles posing systemic risks to human security and socioecological systems ^1–5^. The 2000 megaflood, which was a catastrophic event, resulted in 800 fatalities and inundations of thousands of hectares of agricultural land across Cambodia and Vietnam ^5,6^. These perturbations disproportionately impact over 20 million residents in the riparian zone who work in the flood recession agriculture and capture fishery industries; these peoples’ livelihoods are highly sensitive to stage variability across discharge regimes ^7–11^.

Floods in the LM river basin have undergone pronounced changes since 1950, with significant increases in the frequency and magnitude of floods ^12^. Anthropogenic modifications, including dams, infrastructure (interbasin transfers and diversions) and similar disturbances (land use changes, digging, and sand extraction), have fundamentally altered the seasonal variations in the water level ^13–16^. Moreover, since 2008, the frequencies and magnitudes of floods at different locations in the LM River have declined to varying degrees as anthropogenic modifications increased, with the greatest decline occurring in the upper reaches ^16^. Analyses of climate models have shown that precipitation should increase in the river basin, and the flood flow, water level and inundated area should increase as well, which indicates an increase in the flood risk ^17–22^. Moreover, reservoir operation (i.e., flood control and dry season augmentation) reduces downstream flooding and increases water levels under drought conditions to varying degrees, but it may not be sufficient to offset the increase in flooding caused by climate change ^16–19^. Therefore, effective assessment of future flood‒drought risk requires a comprehensive understanding of the hydrogeomorphic relationship of the LM River under changing conditions.

Despite the urgent need to understand the hydrogeomorphic characteristics along the main stream of the LM River, the morphological effects on stage variability and the different effects of reservoirs under extreme flow regimes are not clear as those under data-scarce fluvial systems (see Supplementary Table S1 online). For example, Chua and Lu ^23^ analysed the water level change due to channel alternation during the dry season and wet season, and the analysed water level change considered the gap between the two stage curves over the entire range of discharge. The discharge range during wet season is indicated by the minimum discharge and maximum discharge from June to November, while the range during dry season is indicated by the minimum discharge and maximum discharge from December to May. In addition, the scholars focused only on Chiang Saen, Mukdahan and Pakse, which is insufficient for describing the effects of climate change and human activities on stage variation in the main stream of the river reach. However, the impacts of climate change and reservoir operation on the flow regime differ spatially and monthly along the main stream ^1,24–26^. For example, climate change increases the variability of preflood streamflow (with a maximum increase in magnitude of 18%), while reservoir operations replenish dry season flows (flow from July to November, with magnitudes decreasing from 34.5 to 36% at 660 km) and reduce extremely high pulses (flow from January to May, with magnitudes decreasing from − 9.5 to − 8% at 660 km) ^24^. Therefore, understanding the hydrogeomorphic relationship (the contribution of channel geometry adjustment to stage variance under extreme flow regimes and the different effects of reservoirs) along the main stream of the LM River can benefit the management and risk prediction of water resources under low- and high-flow conditions, which are key for adaptive watershed management ^27–29^.

Stage–discharge rating curves (RCs) serve as critical hydrodynamic indicators for channel geometry adjustment ^30,31^. Their temporal evolution provides diagnostic evidence of cumulative geomorphic change ^32,33^. In data-scarce systems such as the LM River ^27^, RC analysis offers a proxy approach for quantifying morphological controls of stage variability ^34,35^. This study is aimed at partitioning hydrological and geomorphic contributions to stage variation across discharge regimes (extreme flow regimes during high-flow and low-flow conditions) in the LM River. First, the studied reach of the LM river and the hydrological data (water level and discharge) from the five selected hydrological stations are presented. Second, a method for exploring the contributions of changes in discharge and channel geometry adjustments to stage variation is presented. Finally, the contributions of changes in discharge and channel geometry adjustments to stage variations in the extreme flow regime under high-flow and low-flow conditions are analysed and discussed.

## Study area, data and methodology

### Studied river reach

The length of the main stream of the LM River is 4909 km ^36^. This river originates on the Qinghai‒Tibet Plateau, and it runs through China, Myanmar, the Lao People’s Democratic Republic (Lao PDR), Thailand, Cambodia, and Vietnam before flowing into the South China Sea. This monsoonal system exhibits extreme hydrologic seasonality ^37^, and the annual discharge varies considerably ^17^.

**Fig. 1:**
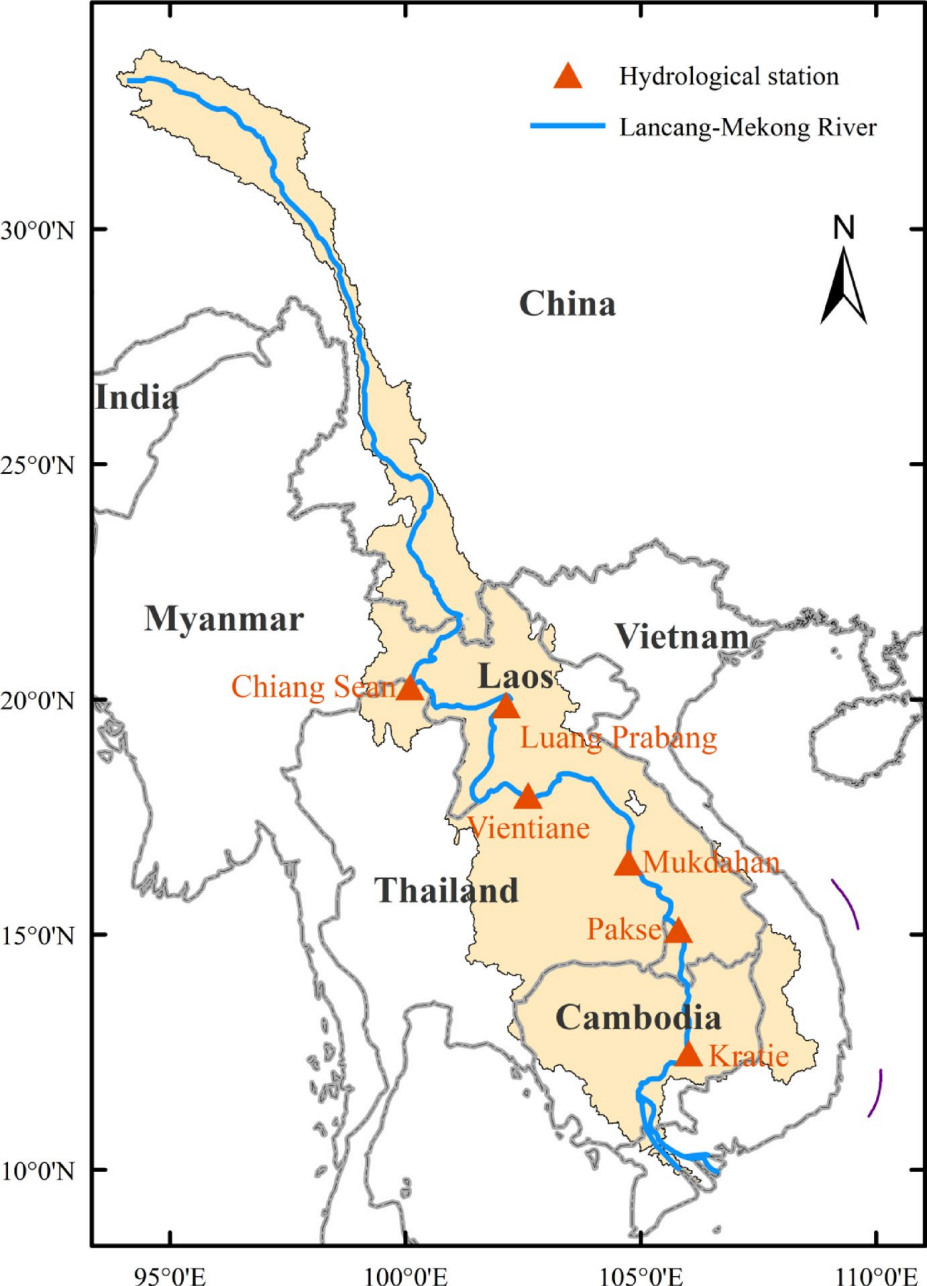
Hydrological stations along the main stream of the LM River.

Five benchmark hydrological stations along the main stream of the LM River were selected (Fig. 1) on the basis of longitudinal representativeness and data continuity (> 60 year daily records with < 7% missing values). Chiang Saen is located on the Thai–Myanmar border. The Chiang Saen–Luang Prabang reach and Luang Prabang–Vientiane reach constitute the upper part of the Mekong River, whereas the Vientiane–Mukdahan reach and Mukdahan–Pakse reach constitute the middle reaches.

### Data

Hydrometric time series (daily discharge and water level) spanning 1960–2020 were acquired from the Mekong River Commission (MRC) (https://portal.mrcmekong.org/) for the five benchmark stations (Chiang Saen, Luang Prabang, Vientiane, Mukdahan and Pakse) (Table 1, see Supplementary Fig. S1 online). The dataset exhibits two temporal discontinuities: Luang Prabang records were discontinued after 2018, and Vientiane observations were unavailable in 2016. The consistency and accuracy of water level data were checked by the MRC ^38^. This dataset has been widely employed to analyse the geographical and hydrologic conditions in relevant river regions (see Supplementary Table S1 online) ^12,39^.

**Table 1:** Data series at the five hydrological stations. See the station locations in Fig. 1.

Station Name	Latitude	Longitude	Basin coverage		Distance to river mouth (km)	Record used	
			(× 10^3^ km^2^)	(%) of Total basin ratio		Discharge	Water level
Chiang Saen	20.274	100.089	199	21	2364	1960–2020	1960–2020
Luang Prabang	19.893	102.134	288	31	2010	1960–2018	1960–2018
Vientiane	17.881	102.632	323	34	1580	1960–2016	1960–2016
Mukdahan	16.583	104.733	429	46	867	1960–2020	1960–2020
Pakse	15.100	105.813	621	66	545	1960–2020	1960–2020

Changing the location of hydrological stations leads to inconsistencies in water level data. Thus, the water level measured at Chiang Saen after 15 December 1993 should have been reduced by 0.62 m after the hydrological station Chiang Saen was moved in 1993 ^40^. In addition, the water level data measured during September and October 2006 at Chiang Saen were notably abnormal ^41^, and the data measured during this period were removed. The water level data drawn from this website were above the corresponding local datum, and these data were modified to metres above sea level according to the values provided by the MRC http://ffw.mrcmekong.org/.

The LM river basin has undergone phased reservoir development. The cumulative storage capacity before 1992 was rather small (for example, the Manwan reservoir was commissioned on 30 June 1993). Until 2007 (for example, the Dachashao reservoir was commissioned in 2003), the total capacity of reservoirs was limited (only 20 km^3^), and the cumulative storage capacity accounted for only 2% of the annual discharge at Pakse ^16,42^. After 2008 (for example, the Xiaowan reservoir was commissioned in September 2009, the Nuozhadu reservoir was commissioned in September 2012, the Xayaburi reservoir was commissioned in 2019, and the Don Sahong hydropower plant was commissioned in 2019), the cumulative storage capacity increased to 400 km^3^ in 2015, accounting for 22% of the annual runoff at Pakse ^38,42–45^. Therefore, in this study, the discharge series was divided into three different periods to compare the effects of reservoirs (see Supplementary Fig. S2 online): the reference period (Period I, pre-1992), the low-intensity disturbance period (Period II, 1993–2007) and the high-intensity disturbance period (Period III, 2008–2020).

### Methodology

The RC methodology served as a critical proxy for reconstructing channel morphodynamics in data-scarce fluvial systems ^46^. This approach has been successfully implemented for flood risk ^47^ and water resource management ^48^. To quantify multidecadal stage variations under evolving channel geometry, the RC form was as follows:

1 $$WL = aQ^{b} + c$$

where *WL* is the water level (m); *Q* is the discharge (m^3^/s); and parameters *a*, *b*, and *c* are coefficients estimated via the nonlinear method in the MATLAB curve fitting toolbox. The coefficient of determination is denoted as *R*^2^.

**Fig. 2:**
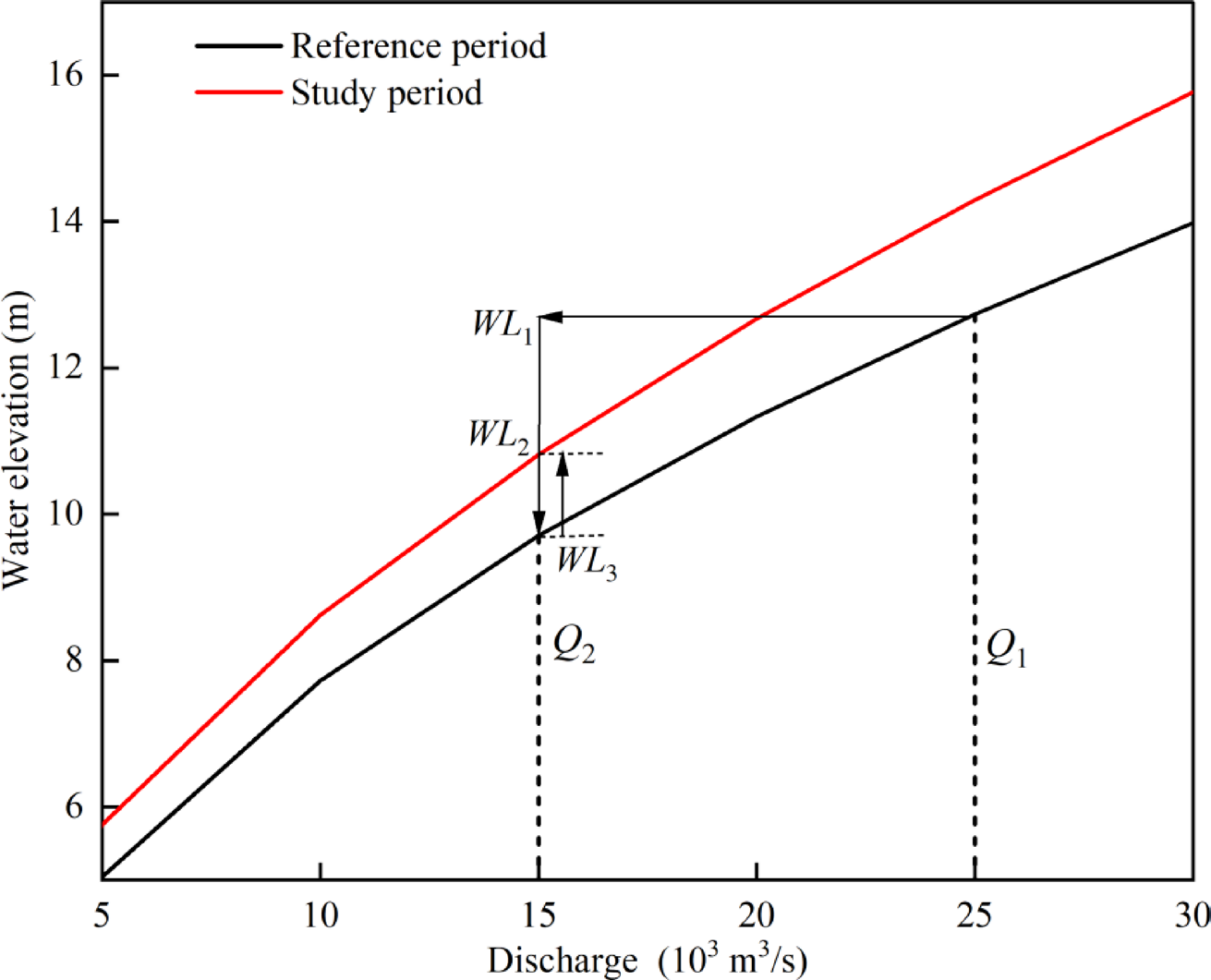
Schematic diagram of the method used to determine the change in water level. Q1 and Q2 are the average values in the reference and study periods, respectively. Under high-flow conditions, Q1 and Q2 are the averages of the extreme flow regime. Under low-flow conditions, Q1 and Q2 are the averages of the extreme flow regime. WL3–WL1 is the change in water level associated with the change in discharge, where a negative value indicates a decrease in the water level as the discharge level decreases. WL2–WL3 is the change in water level caused by a changed channel geometry, and a positive value indicates an increase in the water level caused by channel geometry adjustment. The change in RC is a combined effect of discharge and channel geometry adjustment.

Following the method of Mei et al. ^49^, the stage variation during each period compared with the previous period was analysed. As shown in Fig. 2, *Q*_1_is the value of discharge during the reference period, and *Q*_2_ is the value of discharge during the study period. For the reference period, *WL*_1_ is the water level of *Q*_1_, whereas *WL*_3_ is the water level of *Q*_2_. For the study period, *WL*_2_ is the water level of *Q*_2_. Thus, *WL*_3_–*WL*_1_ is the change in water level associated with the change in discharge (i.e., the discharge decreases from *Q*_1_ to *Q*_2_), with a negative value indicating a decrease in the water level as the discharge decreases. *WL*_2_*–WL*_3_ is the change in water level associated with changes in channel geometry (the same discharge of *Q*_2_ with a changed RC), where a positive value indicates an increased water level due to channel geometry adjustment. Thus, *WL*_2_–*WL*_1_ indicates a decreased water level as a combined effect of changes in both discharge and channel geometry.

The 1-day maximum (${\text{Q}}_{1\text{d}}$) and 90-day maximum (${\text{Q}}_{90\text{d}}$) discharges were chosen as the extreme flow regimes during high-flow conditions, and the 1-day minimum (${\text{Q}}_{1\text{m}}$) and 90-day minimum (${\text{Q}}_{90\text{m}}$) discharges were chosen as the extreme flow regimes under low-flow conditions ^33,45^. The values in each period were calculated as follows:

2 $${\text{Q}}_{1d} = \frac{1}{N}\mathop \sum \limits_{k = 1}^{N} Q_{1k} ,Q_{1k} = {\text{max}}\left\{ {Q_{i,k} } \right\}\left( {{\text{i}} = 1, \ldots ,365} \right)$$

3 $${\text{Q}}_{1m} = \frac{1}{N}\mathop \sum \limits_{k = 1}^{N} Q_{1km} ,Q_{1km} = {\text{min}}\left\{ {Q_{i,k} } \right\}\left( {{\text{i}} = 1, \ldots ,365} \right)$$

4 $${\text{Q}}_{90d} = \frac{1}{N}\mathop \sum \limits_{k = 1}^{N} Q_{90k} ,{\text{Q}}_{90k} = {\text{max}}\left\{ {Q_{90,i,k} } \right\}$$

5 $${\text{Q}}_{90m} = \frac{1}{N}\mathop \sum \limits_{k = 1}^{N} Q_{90km} ,{\text{Q}}_{90km} = {\text{min}}\left\{ {Q_{90,i,k} } \right\}$$

6 $${\text{Q}}_{90,i,k} = \frac{1}{90}\mathop \sum \limits_{i}^{i + 90} Q_{i,k} \left( {{\text{i}} = 1, \ldots ,275} \right)$$

where ${Q}_{i,k}$ indicates the measured discharge of the $i$–th day in the $k$–th year, the subscript *d* indicates the *maximum* value, the subscript *m* indicates the *minimum* value, the subscript *k* indicates the *k-*th year, and $N$ indicates the number of years.

## Results

### Hydrological variation

**Fig. 3:**
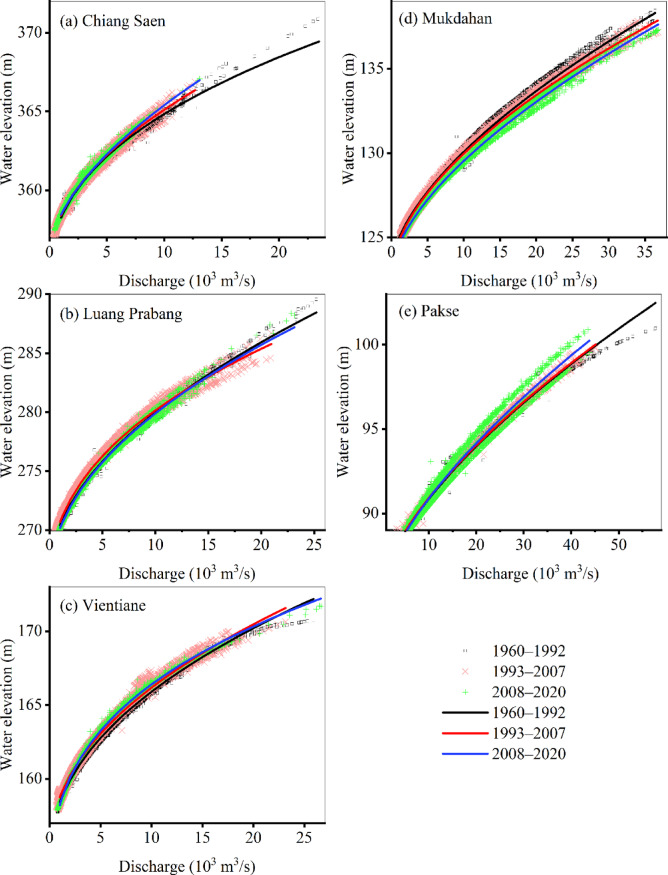
RCs at the hydrological stations Chiang Saen (**a**), Luang Prabang (**b**), Vientiane (**c**), Mukdahan (**d**) and Pakse (**e**) during Periods I, II, and III.

The RCs for the five hydrological stations exhibited spatiotemporal divergence across the three periods (Fig. 3; the parameters are shown in Supplementary Table S2 online). The hysteretic RC shifts revealed specific hydrological responses to disturbances. At the same water level, the discharge levels at Chiang Saen and Pakse were the highest in Period III and lowest in Period I, whereas Mukdahan exhibited the opposite pattern. Moreover, the Luang Prabang and Vientiane stations showed different water level‒discharge relationships between the study periods (with the highest discharge for a given depth occurring in Periods I and II, respectively), confirming that the RCs differed in space and time between the LM reaches.

**Fig. 4:**
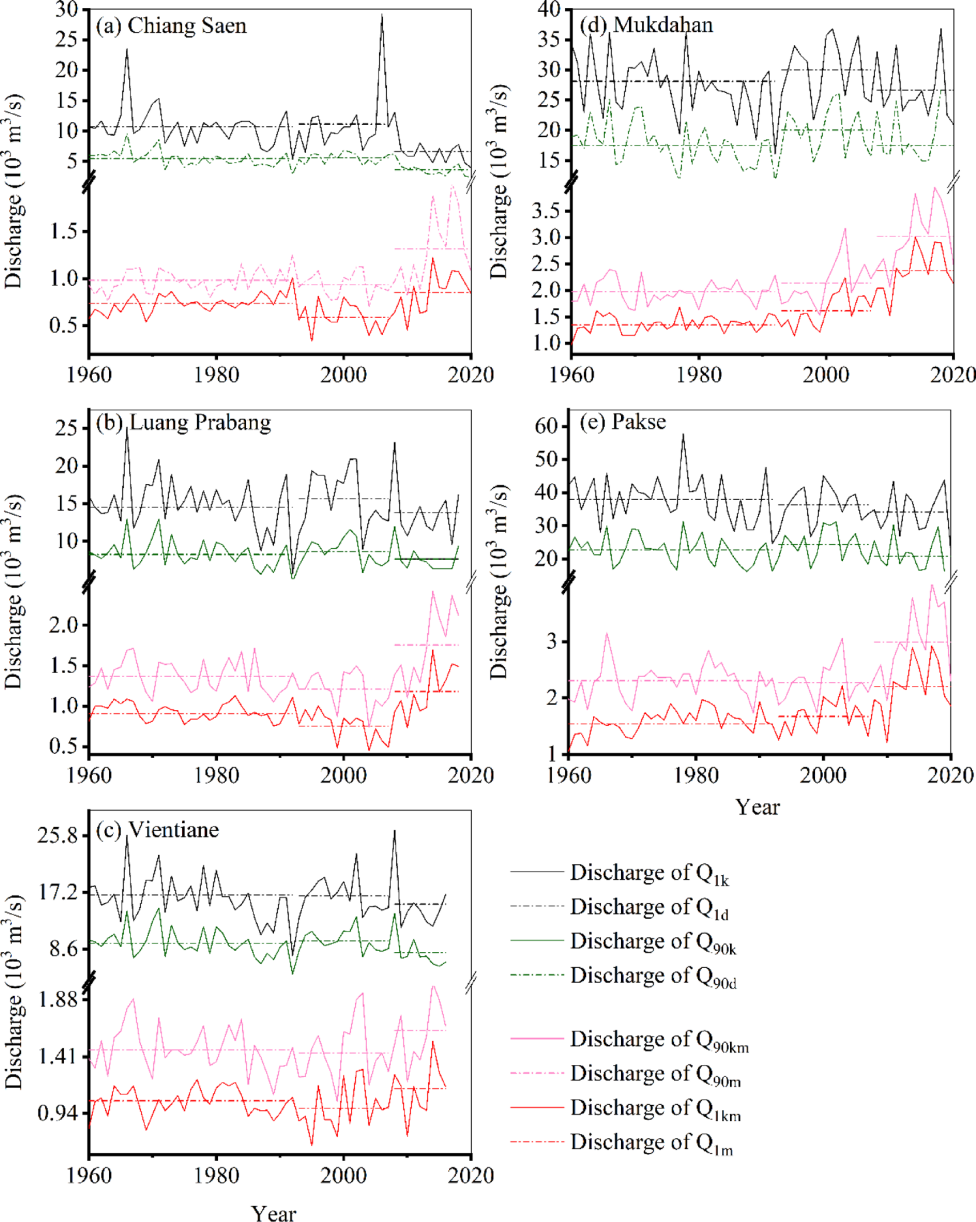
Extreme flow regime under high-flow and low-flow conditions from 19,602,020 at hydrological stations: Chiang Saen (**a**), Luang Prabang (**b**), Vientiane (**c**), Mukdahan (**d**) and Pakse (**e**). The dashed lines are the mean discharges (${\text{Q}}_{1\text{d}}$, ${\text{Q}}_{90\text{d}}$, ${\text{Q}}_{1\text{m}}$ and ${\text{Q}}_{90\text{m}}$) for Periods I, II, and III.

The spatiotemporal evolution of the extreme flow regime (high-flow and low-flow conditions) revealed contrasting responses across the LM River (Fig. 4, see Supplementary Table S3 online**)**. The ${\text{Q}}_{1\text{d}}$ and ${\text{Q}}_{90\text{d}}$ discharges at Chiang Saen, Vientiane and Pakse decreased significantly after 2007 (− 8 ~  − 38%), whereas the discharges of ${\text{Q}}_{1d}$ and ${\text{Q}}_{90d}$ at Luang Prabang and Mukdahan remained relatively unchanged after 1992 (− 5 ~ 8%). The ${\text{Q}}_{1\text{m}}$ and ${\text{Q}}_{90\text{m}}$ discharges along the Chiang Saen–Pakse reach increased significantly after 2007 (9% ~ 80%). Lu and Chua ^41^ reported similar results. Our results suggested flow regime homogenization in the LM River as the difference in the extreme flow regime between the high-flow (maximum) and low-flow (minimum) conditions decreased, especially in the river reach around Chiang Saen.

### Contribution to water level changes

**Fig. 5:**
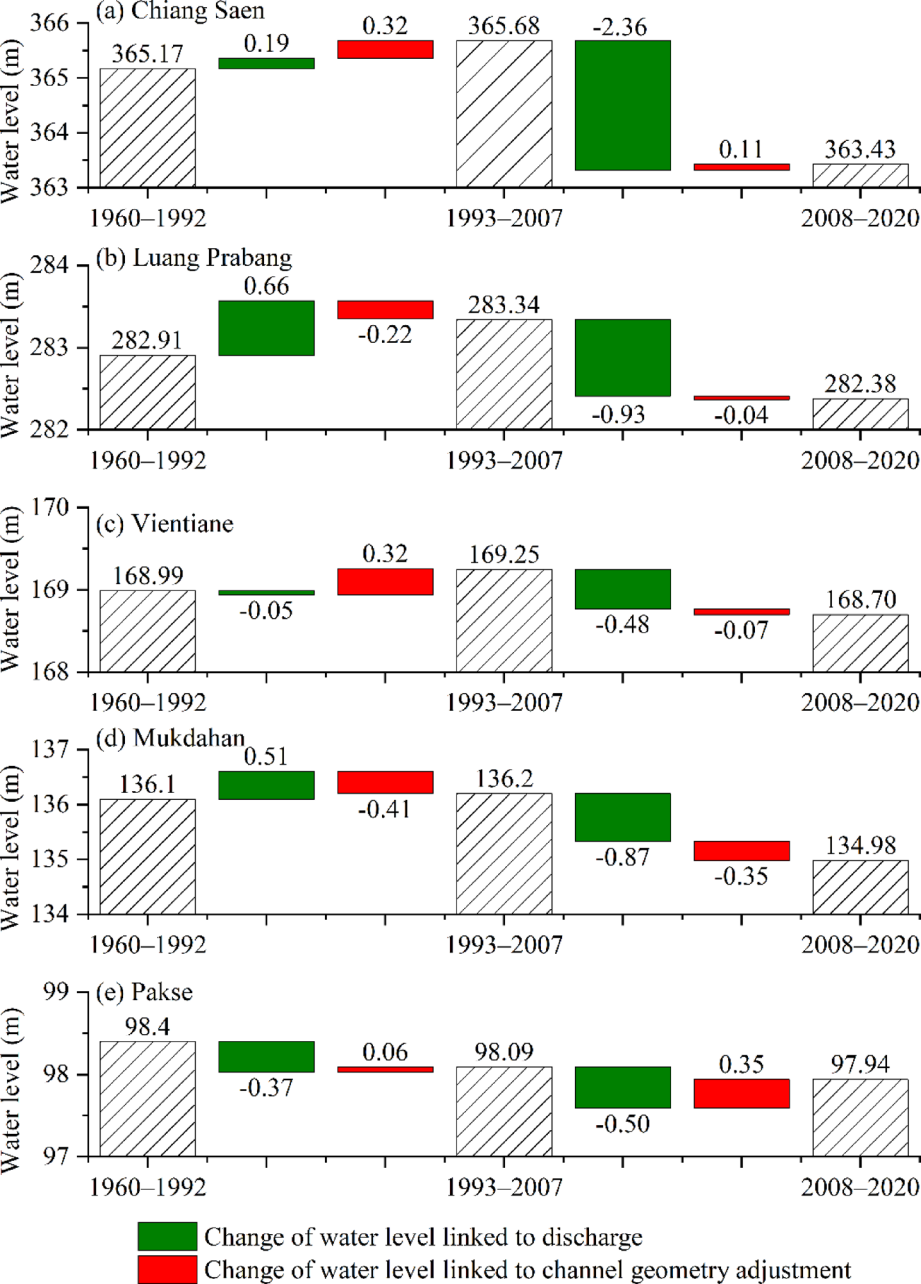
Changes in the water levels for ${\text{Q}}_{1\text{d}}$ at Chiang Saen (**a**), Luang Prabang (**b**), Vientiane (**c**), Mukdahan (**d**) and Pakse (**e**) during Periods I, II and III. The dashed bars are the corresponding water levels of ${\text{Q}}_{1\text{d}}$ during Periods I, II and III. The water level change is divided into contributions by discharge (indicated by green bars) and channel geometry adjustment (indicated by red bars). The numbers above/below the bars are the values of the water level or water level change.

The hydrogeomorphic attribution framework (Fig. 5) quantifies the relative contribution of discharge versus channel geometry adjustment to stage variations in ${\text{Q}}_{1\text{d}}$. The contributions to stage variations for ${\text{Q}}_{90\text{d}}$, ${\text{Q}}_{1\text{m}}$ and ${\text{Q}}_{90\text{m}}$ are summarized in Supplementary Table S4. The analyses below are based on the calculated water levels.

The contribution to stage variation by channel geometry adjustment at the Chiang Saen–Pakse reach varies in the ranges of − 0.57 ~ 0.27 m under low-flow conditions and − 0.41 ~ 0.39 m under high-flow conditions. In comparison, the stage variation linked to discharge varies in the ranges of − 0.37 ~ 1.3 m under low-flow conditions and − 2.36 ~ 0.66 m under high-flow conditions (see Supplementary Table S4 online). This finding indicates that stage variations along the main stream of the LM River under both high-flow and low-flow conditions are caused mainly by discharge.

**Fig. 6:**
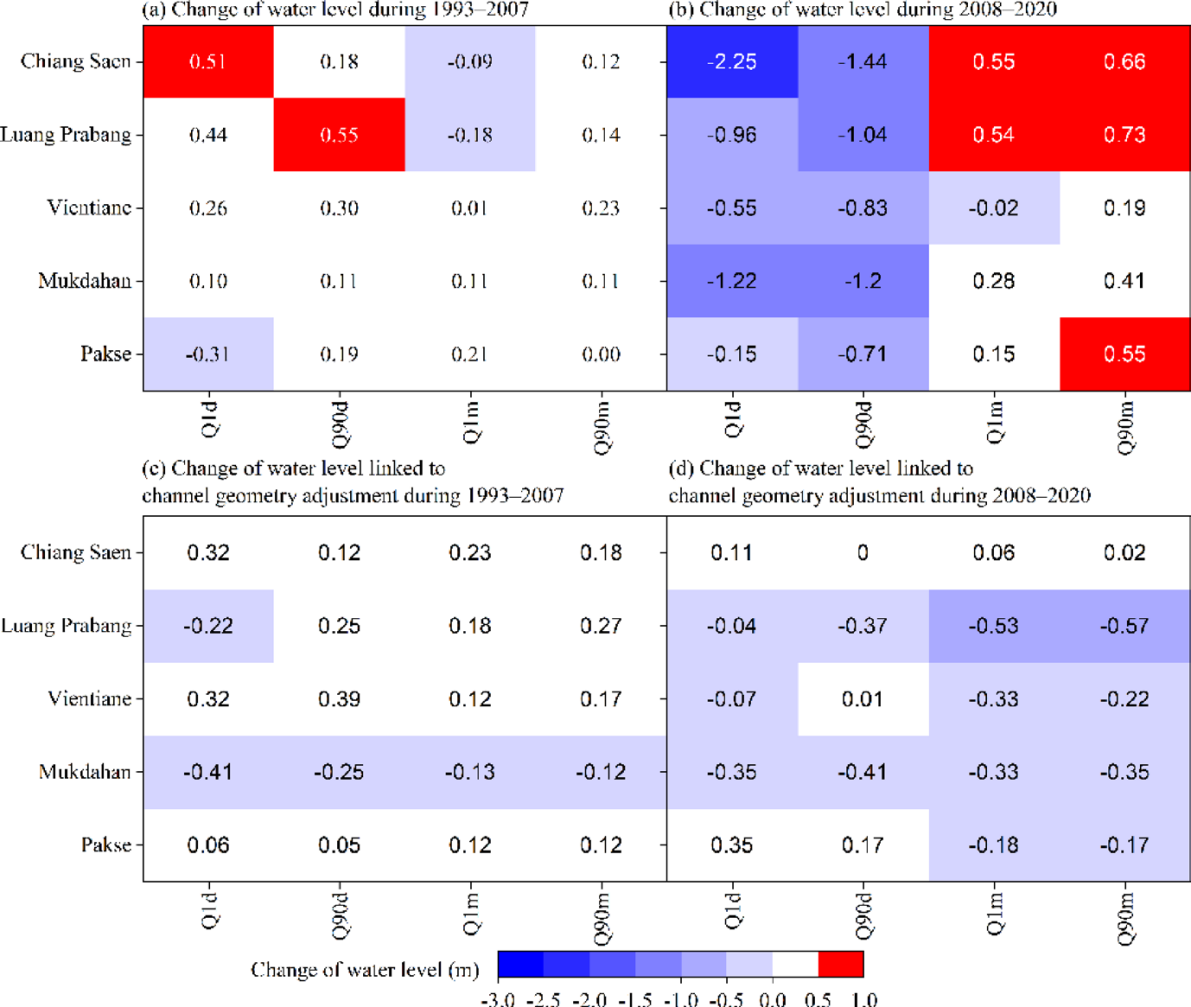
Heatmap of changes in water level under high-flow and low-flow conditions in Periods II and III. Changes in the combined (i.e., influenced by both discharge and channel geometry adjustment) water level (**a** and **b**) and changes in the water level linked to channel geometry adjustment (**c** and **d**) at the five hydrological stations are shown.

The spatiotemporal patterns of geometry-driven stage adjustments reveal three distinct response regimes (Fig. 6).

The stage variation ranges linked to channel geometry adjustment under low-flow conditions at these five hydrological stations are 0.02 ~ 0.23 m for Chiang Saen, − 0.57 ~ 0.27 m for Luang Prsabang, − 0.33 ~ 0.17 m for Vientiane, − 0.35 ~ –0.12 m for Mukdahan, and − 0.18 ~ 0.12 m for Pakse, whereas the corresponding ranges under high-flow conditions are 0 ~ 0.32 m for Chiang Saen, − 0.37 ~ 0.25 m for Luang Prabang, − 0.07 ~ 0.39 m for Vientiane, − 0.41 ~  − 0.25 m for Mukdahan, and 0.05 ~ 0.35 m for Pakse. Channel geometry adjustments under high-flow and low-flow conditions after 1992 increase the water level in the river reach around Chiang Saen and decrease the water level in the river reach around Mukdahan (Fig. 6c and d). The results suggest that the river reach of Luang Prabang–Vientiane is a transitional reach in terms of the effects of channel geometry adjustment on water level changes. Specifically, under both low-flow and high-flow conditions, the water level linked to channel geometry adjustment at Vientiane is positive during Period II (Fig. 6c), whereas it is negative during Period III (Fig. 6d). This finding indicates that the effects of reservoirs on the variation in water level are linked to channel geometry adjustment.

Overall, discharge is a dominant factor influencing stage variation at the five hydrological stations under both high-flow and low-flow conditions (Fig. 6, see Supplementary Table S4 and Supplementary Fig. S3 online), whereas the contribution of channel adjustment modulates it. The proportion of channel geometry adjustment that corresponds to a counteractive adjustment of stage variation differs under different conditions, with values of 60% and 40% under low-flow and high-flow conditions, respectively.

## Discussion

Our analyses show the modulation of channel geometry adjustment to stage variation along the main stream of the LM River under both high-flow and low-flow conditions. The uncertainties and implications are discussed in this section.

### Uncertainties

Stage variability emerges from the coupled effects of discharge and channel morphodynamics ^32^. RC extrapolation is associated with uncertainties due to multiple sources of error (17 ~ 37% for automated river gauges) ^28,46,50–52^. The uncertainties of RCs are greater during flood events ^27,53^. For example, the RCs for floods lasting several days vary during the rising and falling periods. However, the uncertainties are minimized by the use of reliable daily discharge data. The daily discharge data analysed here are estimated on the basis of updated RCs ^54,55^, and the quality of the data is assured ^56^. The daily calculated discharge has been considered the observed discharge when measured data are not available. For example, Räsänen et al. ^56^ used the calculated discharge at Chiang Saen for the period 1960–1990 as the observed discharge. Uncertainties can be partially examined by comparing the calculated water levels with the measured values (Fig. S4). The difference between the calculated and observed water levels is limited, and the difference under high-flow conditions (in the range of − 0.09 ~ 0.14 m) is greater than that under low-flow conditions (in the range of − 0.03 ~ 0.06 m). Importantly, the relative errors induced by the RC curve uncertainties are considerably smaller than the magnitudes of the changes in the ${\text{Q}}_{1\text{d}}$ and ${\text{Q}}_{90\text{d}}$ water levels. For example, the change in the water level for ${\text{Q}}_{1\text{d}}$ at Chiang Saen under high-flow conditions after 2010 is − 2.25 ~  − 1.24 m, and the value for ${\text{Q}}_{90\text{d}}$ at Chiang Saen under high-flow conditions from 2008 to 2020 is − 1.55 ~  − 1.44 m; these values are variable but considerably larger than the RC uncertainty values in this study and in the literature ^23,41^.

With many more dams being constructed in the future ^57,58^, RCs should be updated accordingly with field surveys to make more accurate discharge estimates and minimize the influences of these uncertainties. And the satellite-derived morphology and flume experiments should be analyzed to directly address morphology-stage linkages.

### Implications for high-flow conditions

The effects of channel geometry adjustments on changes in water levels can be discussed even with these uncertainties.

Under high-flow conditions, the positive change in water level along the Chiang Saen–Mukdahan reach in Period II switches to a negative change in Period III (Fig. 6). The positive change in water level that is linked to discharge along Chiang Saen–Luang Prabang under high-flow conditions (0.06 ~ 0.66 m in Period II) is transformed into a negative change (− 2.36 ~ 0.93 m in Period III). Moreover, the changes in water levels under low-flow conditions (${\text{Q}}_{1\text{m}}$) linked to channel geometry adjustments along the Luang Prabang–Vientiane reach, which vary from positive (0.12 ~ 0.18 m in Period II) to negative (− 0.53 ~  − 0.33 m in Period III), follow the same pattern. A sharp decrease in discharge at Chiang Saen under high-flow conditions in Period III (larger than 30%) indicates that the stream power and sediment load are reduced because of the effects of upstream dams. There is a corresponding reduction in the effects on channel geometry adjustment. The limited decrease in discharge along Luang Prabang–Mukdahan under high-flow conditions in Period III (less than 10%) indicates that the effects of sediment trapping and discharge attenuation are reduced by upstream dams. Our results indicate that channel geometry adjustment has a modulating effect, with the exception of the effect of discharge on water level changes, which decreases in the reach around Luang Prabang and almost disappears in the reach downstream at Vientiane.

Erosion in the Chiang Saen–Mukdahan reach under high-flow conditions is related to the differences in the suspended sediment loads compared with those under mean conditions. For example, the suspended sediment loads around the Chiang Saen River reach under high-flow conditions in Period I range from 350 to 1200 mg/L, whereas the values decrease during Period II ^59,60^.

**Fig. 7:**
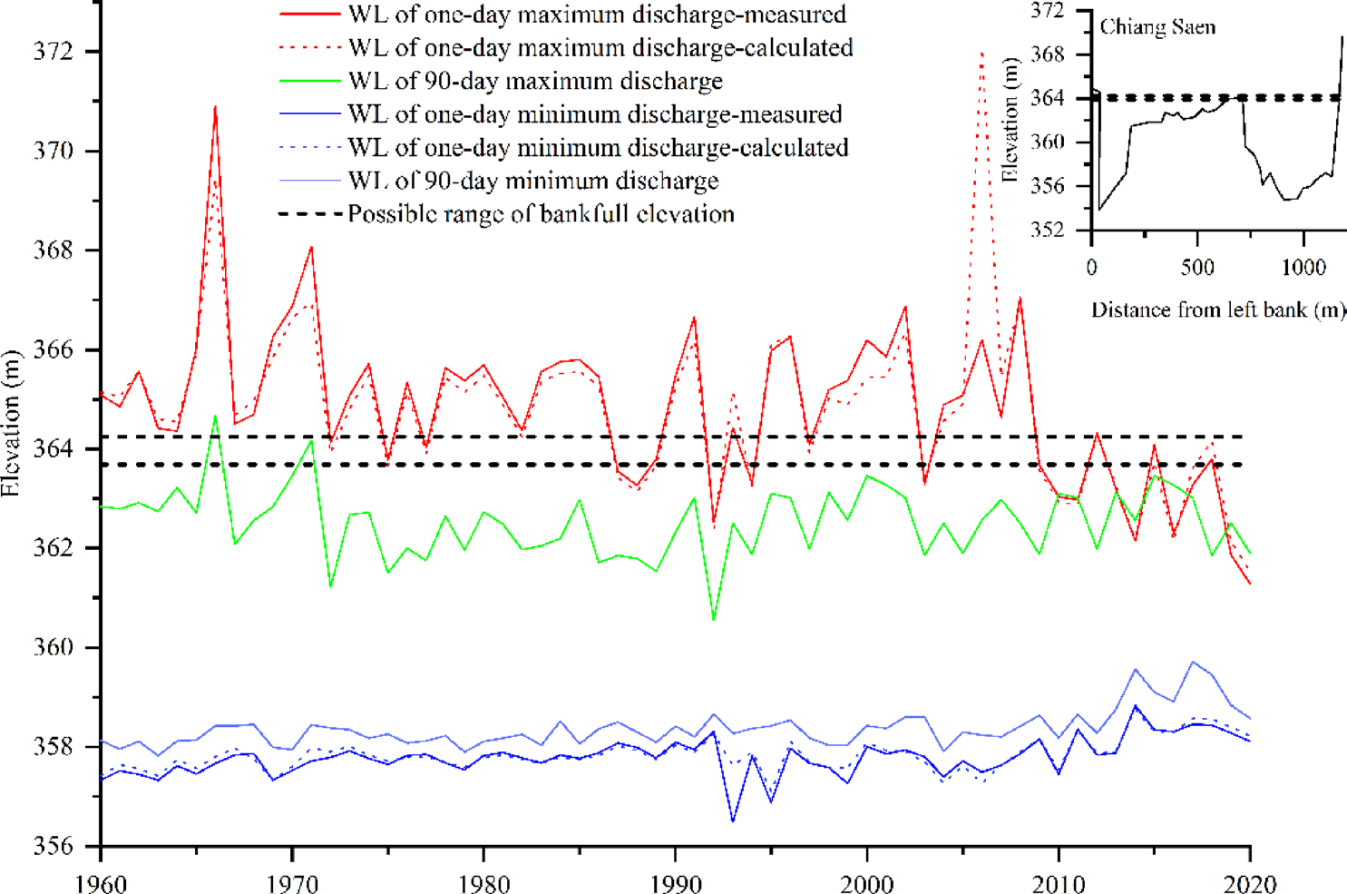
Water level and threshold of bank full elevation at Chiang Saen. For ${\text{Q}}_{1\text{d}}$ and ${\text{Q}}_{1\text{m}}$, both the measured and calculated water levels are illustrated, whereas only those calculated for ${\text{Q}}_{90\text{d}}$ and ${\text{Q}}_{90\text{m}}$ are illustrated. The bankfull elevation are estimated by 1.5 return period discharge (363.89–364.25 m). The cross-sectional data are drawn from Hou, S.Y. (2021).

In addition to changes in channel geometry adjustment (e.g., riverbed erosion and deposition and bank erosion) and stream power ^61^, geometric constraints may contribute to the effects of channel geometry adjustment on changes in the water level ^62–65^. For the river cross section at the Chiang Saen hydrological station (Fig. 7, see Supplementary Fig. S5 online, cross-sectional data from Hou 2021 ^37^), the water levels for ${\text{Q}}_{1\text{d}}$ in Periods I and II are higher than those of the bankfull elevation (water stage of 1.5 return period discharge ^66^); these characteristics differ from those of Period III (Fig. 7). There are also planform changes in the river reach around hydrological stations (see Supplementary Fig. S6 online), and this planform change in the river reach also contributes to the stage variation as well ^67^.This finding indicates that at Chiang Saen, the hydrodynamic properties of floods and the effects of channel geometry adjustments on water level changes differ during Period III.

### Implications for low-flow conditions

**Fig. 8:**
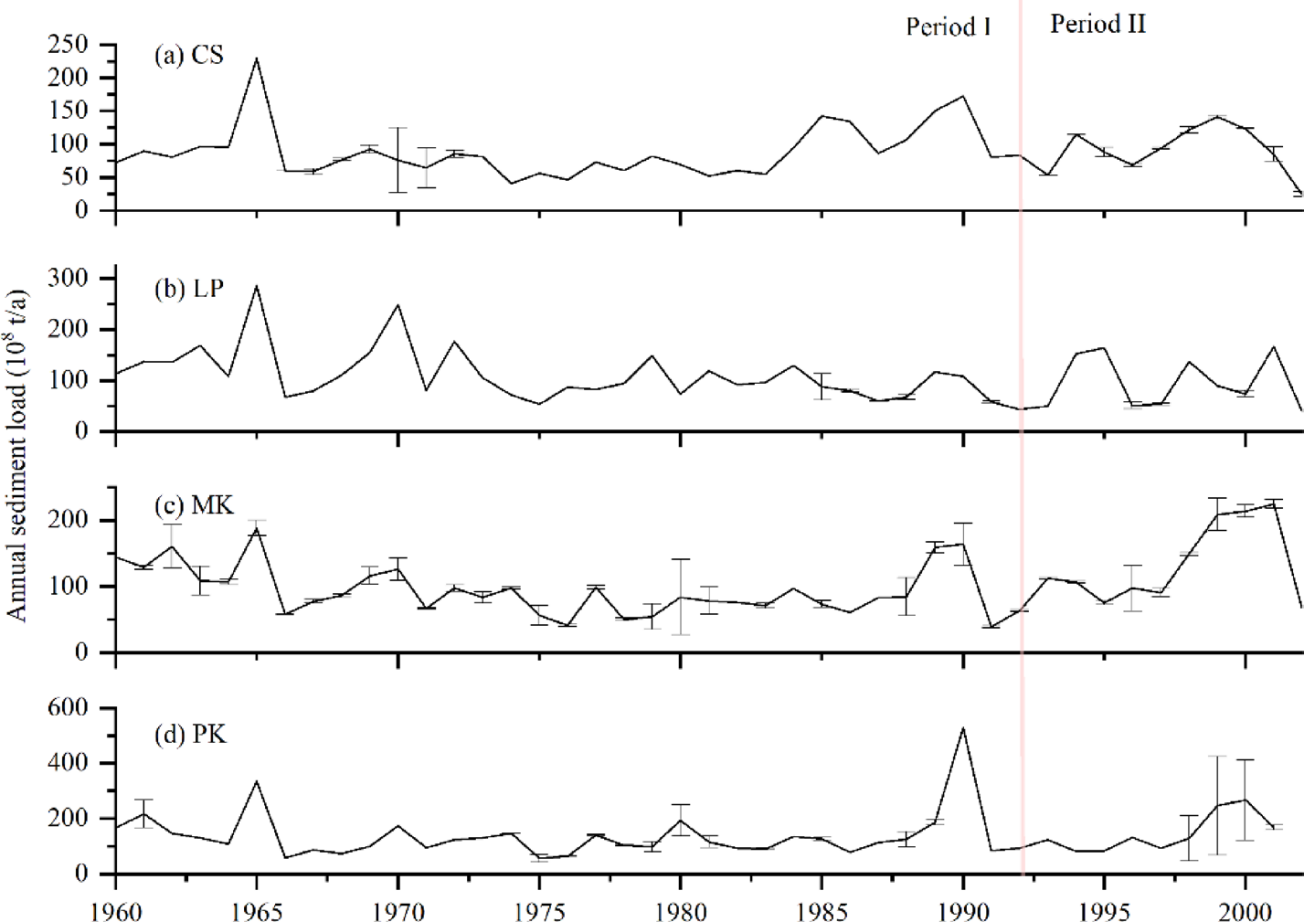
Annual sediment load along the LM River. The data are drawn from Walling (2008), Wang et al. (2011), and Liu et al. (2013). The annual sediment load at Chiang Saen (increased by 4%), Mukdahan (44%), and Pakse (7%) increased from 1993–2003, whereas the value at Luang Prabang (− 11%) decreased.

Water levels for ${\text{Q}}_{1\text{m}}$ under low-flow conditions in Period II decrease along the Chiang Saen–Luang Prabang reach (− 0.09 m for Chiang Saen and − 0.18 m for Luang Prabang) and increase along the Vientiane–Pakse reach (0.01 m for Vientiane, 0.11 m for Mukdahan, and 0.21 m for Pakse). An increase in the annual sediment load along the Mukdahan–Pakse reach (Fig. 8; data from Wang 2008 ^68^, Wang et al. 2011 ^69^ and Liu et al. ^70^) leads to a positive change in water levels along the Mukdahan–Pakse reach in Period II. For the reach around Vientiane, the erosion of river islands (near the Vientiane hydrological station) is severe (0.6 m/a for 1961–1992 and 6.4 m/a for 1992–2005) ^71^. This severity results in increased stream power at Vientiane, which attenuates the effects of a small decrease in ${\text{Q}}_{1\text{m}}$ (− 6%, see Supplementary Table S3 online) in Period II and leads to a limited increase in water levels (0.01 m) under low-flow conditions. Similarly, there are sediment-contributing areas in the Chiang Saen–Luang Prabang reach in Periods I and II due to the expansion of cultivated land and plantations ^70^. However, the effects of decreased ${\text{Q}}_{1\text{m}}$ (− 20%, see Supplementary Table S2 online) for the reach around Chiang Saen outweigh the increased annual sediment load (Fig. 8a) in Period II. For the reach around Luang Prabang, the effects of decreased ${\text{Q}}_{1\text{m}}$ (− 17%, see Supplementary Table S3 online) lead to a large change in water level (− 0.18 m), whereas the related channel geometry adjustments include the erosion of islands and bars as the LM River has a stable bank line ^71^. This finding suggests that the effects of channel geometry adjustment downstream from the Vientiane hydrological station (namely, the Vientiane–Pakse reach) on the water level offset the effects of increased suspended sediment load. Cochrane et al. ^40^ reported a diminished effect of dams, which is negligible at Vientiane.

Water levels for ${\text{Q}}_{1\text{m}}$ under low-flow conditions from 2008 to 2020 increased along Chiang Saen–Pakse (0.48 ~ 0.56 m for Chiang Saen, 0.54–0.62 m for Luang Prabang, − 0.02 m for Vientiane, 0.28 ~ 0.37 m for Mukdahan, and 0.15 ~ 0.35 m for Pakse). The stage variations in ${\text{Q}}_{90\text{m}}$ along the Chiang Saen–Luang Prabang reach after 1992 are positive: 0.12 ~ 0.66 m for Chiang Saen and 0.14 ~ 0.73 m for Luang Prabang. The water levels under low-flow conditions along the Vientiane–Pakse reach have increased after 1992: − 0.02 ~ 0.23 m for Vientiane, 0.11 ~ 0.41 m for Mukdahan, and 0 ~ 0.55 for Pakse. In total, the stage variation along the Chiang Saen–Pakse reach under low-flow conditions is influenced mainly by discharge (see Supplementary Fig. S3 online), and the channel has a homogenized flow regime (Fig. 4) and reduced erosive capacity (from runoff changes) during the high-intensity disturbance period.

During the high-intensity disturbance period (2008–2020), the change in the water level linked to channel geometry adjustment at Chiang Saen (0 ~ 0.11 m) was limited. Under low-flow conditions, the change in the water level of ${\text{Q}}_{90\text{m}}$ discharge at Chiang Saen during the low–intensity disturbance period (1993–2007) was variable at + 0.12 ~ 0.202 m, whereas the change in the water level of ${\text{Q}}_{1\text{m}}$ discharge during the high-intensity disturbance period (2008–2020) was 0.48–0.56 m, as shown in the literature and this study ^41,48^. Moreover, the stage variation in ${\text{Q}}_{1\text{m}}$ discharge linked to channel geometry adjustment was 0.06 m during the high-intensity disturbance period (2008–2020). The reported percentage contribution of channel geometry adjustment to water level change by Lu and Chua ^41^ was 4% at Chiang Saen (3.53 m water level from 20,102,020 versus 3.37 m water level from 19,601,991). Channel geometry adjustment around Chiang Saen station has mainly been caused by new sandbar formation resulting from bedload transport ^59^.

## Conclusions

The aim of this study was to explore the magnitude and range of the contribution of channel geometry adjustment to stage variance along the main stream of the LM River under an extreme flow regime. Therefore, historical daily discharge and water levels at five hydrological stations (Chiang Saen, Luang Prabang, Vientiane, Mukdahan and Pakse) from 1960–2020 were acquired. Four parameters (1-day maximum discharge, 90-day maximum discharge, 1-day minimum discharge, and 90-day minimum discharge) were adopted to represent the historical extreme flow regimes under high- and low-flow conditions. Variations in RCs were adopted to distinguish the effects of discharge and channel geometry adjustment on water level variation. The conclusions were as follows:

1. Overall, under high-flow conditions, the water level along the Chiang Saen–Mukdahan reach decreased between 1960 and 2020. Our analysis revealed that the water level increased between 1993 and 2007 (low-intensity disturbance period) but decreased with increasing magnitude between 2008 and 2020 (high-intensity disturbance period). However, the change in the water level under low-flow conditions varied across different reaches and different flow conditions. Overall, the results differed between the reaches upstream and downstream from the Luang Prabang reach.
2. Our analysis revealed that discharge was the dominant factor influencing stage variance under both high-flow and low-flow conditions along the main stream of the LM River, whereas it was further modulated by channel geometry adjustment. Our analysis showed that the modulation by channel geometry adjustment varied under high-flow and low-flow conditions. The modulation by channel geometry adjustment at minimum discharge varied in the range of − 0.57 ~ 0.27 m (0.02 ~ 0.23 m for Chiang Saen, − 0.57 ~ 0.27 m for Luang Prabang, − 0.33 ~ 0.17 m for Vientiane, − 0.35 ~  − 0.12 m for Mukdahan and − 0.18 ~  − 0.12 m for Pakse). Moreover, the modulation by channel geometry adjustment under high-flow conditions varied in the range of − 0.41 ~ 0.39 m (0 ~ 0.32 m for Chiang Saen, − 0.37 ~ 0.25 m for Luang Prabang, − 0.07 ~ 0.39 m for Vientiane, − 0.41 ~  − 0.25 m for Mukdahan and 0.05 ~ 0.35 m for Pakse).
3. Across the five hydrological stations analysing the four extreme flow regimes, inverse channel geometry adjustment responses (opposing water level trends) were observed in 60% of the high-flow conditions versus in 40% of the low-flow conditions. In addition, the river reach of Luang Prabang–Vientiane was a transitional reach in terms of the effects of channel geometry adjustment on water level changes under an extreme flow regime.

At Chiang Saen, changes in discharge and limited channel geometry adjustment influenced by upstream dams, along with cross-sectional geometry, could contribute to stage variation, and field surveys could further verify these claims. Luang Prabang–Vientiane was considered a transitional reach, as the combined effect (discharge, sediment and sediment–discharge regimes) on the change in water level decreased in the Luang Prabang reach and almost disappeared downstream at the Vientiane reach.

## Supplementary Information

Below is the link to the electronic supplementary material.

Supplementary Material 1.

## Data Availability

Data from the Mekong River Commission (MRC) (https://portal.mrcmekong.org/) were utilized for the analysis in this study.
