# Supplementary material for: Contribution of channel geometry adjustments to stage variance based on rating-curves in the main stream of the Lancang–Mekong river
Source: Sci Rep. 2025 Oct 15;15:35988. doi: 10.1038/s41598-025-19822-w (PMC12528363; doi:10.1038/s41598-025-19822-w)
Supplement: Supplementary file 1 — Supplementary Material 1. [file 41598_2025_19822_MOESM1_ESM.pdf]

Contribution of channel geometry adjustments to stage variance based on rating-curves in the main stream of the Lancang–Mekong River

Li He [1*], Qiuhong Tang [1, 2], Dong Chen [1, 2], Paul P.J. Gaffney [1], Gaohu Sun [3]

[1] Key Laboratory of Water Cycle and Related Land Surface Processes, Institute of Geographic Sciences and Natural Resources Research, Chinese Academy of Sciences, Beijing 100101, China

[2] University of Chinese Academy of Sciences, Beijing 100049, China

[3] International Economic & Technical Cooperation and Exchange Center, Ministry of Water Resources, Beijing 100053, China

Correspondence: Li He (heli@igsnrr.ac.cn)

(supplementary file)

Supplement:

Steps of RC method

Supplementary Table S1: Comparison of changes in water level by channel deformation in the LM River.

Supplementary Table S2: Fitted parameters of rating curves.

Supplementary Table S3: The Q_{1d}, Q_{90d}, Q_{1m} and Q_{90m} discharges at the five hydrological stations.

Supplementary Table S4: Change of water level, change of water level liked to discharge and channel deformation.

Supplementary Figure S1 Bar chart of discharge (Colum a) and water level (Colum b). Water level are data above the corresponding local datum.

Supplementary Figure S2 Temporal variation of total storage capacity of reservoir (a) and anomalies of storage capacity of reservoir (b) by cumulative anomaly method.

Supplementary Figure S3: Contributions of discharge and channel deformation to water level changes.

Supplementary Figure S4. Evaluation of fitted rating curves.

Supplementary Figure S5: Width and channel cross sections of the five hydrology stations.

Supplementary Figure S6 Planform variation of river channel around the five hydrology station. Images are drawn from 2024 and 2000, respectively.

Reference

Steps of RC method.

Take the calculation of water level of the mean 1–day maximum (Q_{1d}) discharge at Chiang Saen as an example to elucidate the RC method.

The first step, fit the RC curves of the two periods (1960–1992, 1993–2007) based on collected hydrology data, and get corresponding parameters of (a1, b1, and c1) for period of 1960–1992 and (a2, b2, and c2) for period of 1993–2007.

The second step, calculate the discharges of Q1 (Q_{1d} for period of 1960–1992) and Q2 (Q_{1d} for period of1993–2007) at Chiang Saen.

The third step, calculate the WL1 of Q1 (Q_{1d} for period of 1960–1992) and WL3 of Q2 (Q_{1d} for period of 1993–2007) based on fitted parameters of (a1, b1, and c1) in 1960–1992.

The fourth step, calculate the WL2 of Q2 (Q_{1d} for period of 1993–2007) based on fitted parameters of (a2, b2, and c2) in 1993–2007.

The fifth step, calculated WL3– WL1 and WL2– WL3, values are the change in water level associated with the change in discharge and channel deformation, respectively.

Supplementary Table S1. Comparison of changes in water level by channel deformation in the LM River. This study focuses on the response of water level of peak discharges during the dry and wet situations to channel deformation, including the magnitude and range of its contribution on water level change. As the division of the study periods and selection of hydrological stations, it is possible to distinguish the effects of reservoir and the longitudinal reduction of this effect.

Hydrological Station	Remarks	Range of Data	Reference
Chiang Saen	Dry-season (December to May): changes in water level by channel alternation are -0.08 m (1990-1999), 0.20 m (2000-2009), -0.03 m (2010-2019) compared to 1980-1989 (Fig. 6);<br>Wet-season (June to November): water level 0.01 m (1990-1999), 0.11 m (2000-2009), -0.17 m (2010-2019) compared to 1980-1989 (Fig. 6).	Data records of 1980-2019 for water level and discharge, data were analysed in 10-years intervals compared to the 1980-1989	Chua and Lu [1]
	Peak discharges (Q_{1m}, Q_{90m}) during the low-flow situation: changes in water level by channel alternation are 0.18~0.23 m (1993-2007), 0.02~0.06 m (2008-2020) compared to 1960-1992;<br>Peak discharges (Q_{1d}, Q_{90d}) during the high-flow situation: changes in water level by channel alternation are 0.12~0.32 m (1993-2007), 0.00~0.11 m (2008-2020) compared to 1960-1992.	Data records of 1960-2020 for water level and discharge, peak discharges were analysed in the low-intensity disturbance period (1993–2007) and the high-intensity disturbance period (2008–2020) compared to the reference period (before 1992)	In this study
Luang Prabang	Peak discharges (Q_{1m}, Q_{90m}) during the low-flow situation: changes in water level by channel alternation are 0.18-0.27 m (1993-2007), -0.57~-0.53 m (2008-2020) compared to 1960-1992;<br>Peak discharges (Q_{1d}, Q_{90d}) during the high-flow situation: changes in water level by channel alternation are -0.22~0.25m (1993-2007), -0.37~-0.04 m (2008-2020) compared to 1960-1992.	Data records of 1960-2020 for water level and discharge, peak discharges were analysed in the low-intensity disturbance period (1993–2007) and the high-intensity disturbance period (2008–2020) compared to the reference period (before 1992)	In this study
Vientiane	Peak discharges (Q_{1m}, Q_{90m}) during the low-flow situation: changes in water level by channel alternation are 0.12~0.17 m (1993-2007), -0.33~-0.22 m (2008-2020) compared to 1960-1992;<br>Peak discharges (Q_{1d}, Q_{90d}) during the high-flow situation: changes in water level by channel alternation are 0.32~0.39 m (1993-2007), -0.07~0.10 m (2008-2020) compared to 1960-1992.	Data records of 1960-2020 for water level and discharge, peak discharges were analysed in the low-intensity disturbance period (1993–2007) and the high-intensity disturbance period (2008–2020) compared to the reference period (before 1992)	In this study
Mukdahan	Dry-season (December to May): changes in water level by channel alternation are -0.05 m (1990-	Data records of 1980-2019 for water level and	Chua and

	1999), -0.21 m (2000-2009), -0.56 m (2010-2019) compared to 1980-1989 (Fig. 6);<br>Wet-season (June to November): water level -0.08 m (1990-1999), -0.45 m (2000-2009), -0.81 m (2010-2019) compared to 1980-1989 (Fig. 6).	discharge, data were analysed in 10-years intervals	Lu [1]
	Peak discharges (Q_{1m}, Q_{90m}) during the low-flow situation: changes in water level by channel alternation are -0.13~-0.12 m (1993-2007), -0.35~-0.33 m (2008-2020) compared to 1960-1992;<br>Peak discharges (Q_{1d}, Q_{90d}) during the high-flow situation: changes in water level by channel alternation are -0.41~-0.25 m (1993-2007), -0.41~-0.35 m (2008-2020) compared to 1960-1992.	Data records of 1960-2020 for water level and discharge, peak discharges were analysed in the low-intensity disturbance period (1993–2007) and the high-intensity disturbance period (2008–2020) compared to the reference period (before 1992)	In this study
Pakse	Dry-season (December to May): changes in water level by channel alternation are 0.07 m (1990-1999), -0.03 m (2000-2009), -0.18 m (2010-2019) compared to 1980-1989 (Fig. 6);<br>Wet-season (June to November): water level 0.09 m (1990-1999), -0.02 m (2000-2009), -0.04 m (2010-2019) compared to 1980-1989 (Fig. 6).	Data records of 1980-2019 for water level and discharge, data were analysed in 10-years intervals	Chua and Lu [1]
	Peak discharges (Q_{1m}, Q_{90m}) during the low-flow situation: changes in water level by channel alternation are 0.12 m (1993-2007), -0.18~-0.17 m (2008-2020) compared to 1960-1992;<br>Peak discharges (Q_{1d}, Q_{90d}) during the high-flow situation: changes in water level by channel alternation are 0.05~0.06 m (1993-2007), 0.17~0.35 m (2008-2020) compared to 1960-1992.	Data records of 1960-2020 for water level and discharge, peak discharges were analysed in the low-intensity disturbance period (1993–2007) and the high-intensity disturbance period (2008–2020) compared to the reference period (before 1992)	In this study

Supplementary Table S2. Fitted parameters of rating curves during Period I, Period II and Period III at the five hydrological stations.

Parameters	Period I (1960–1992)	Period II (1993–2007)	Period III (2008–2020)	Parameters	Period I (1960–1992)	Period II (1993–2007)	Period III (2008–2020)
	Chiang Saen				Mukdahan		
a	0.217	0.101	0.045	*a*	0.024	0.036	0.024
b	0.422	0.497	0.579	*b*	0.614	0.575	0.612
c	354.250	355.279	355.969	*c*	123.290	122.897	122.916
R^2	0.9929	0.9788	0.988	R^2	0.9959	0.9964	0.998
Parameters	Luang Prabang			Parameters	Pakse		
a	0.112	0.293	0.159	*a*	0.015	0.013	0.012
b	0.522	0.428	0.489	*b*	0.643	0.652	0.665
c	266.325	265.055	265.524	*c*	85.285	85.466	85.264
R^2	0.9961	0.9932	0.9956	R^2	0.9987	0.9990	0.9895
Parameters	Vientiane						
a	0.166	0.287	0.890				
b	0.460	0.411	0.314				
c	154.43	154.88	150.879				
R^2	0.9949	0.9934	0.9988				

Supplementary Table S3. The $\mathbf{Q_{1d}}$, $\mathbf{Q_{90d}}$, $\mathbf{Q_{1m}}$ and $\mathbf{Q_{90m}}$ discharges at the five hydrological stations.

Hydrological Station	Stream parameter	Period I	Period II		Period III	
		Mean discharge (m^3/s)	Mean discharge (m^3/s)	Deviation magnitude (%)	Mean discharge (m^3/s)	Deviation magnitude (%)
Chiang Saen	Q_{1d}	10,752	11,194	442/4	6,673	-4,079/-38
	Q_{90d}	5,745	5,840	95/2	3,809	-1,937/-34
	Q_{1m}	741	590	-151/-20	854	113/15
	Q_{90m}	880	845	-34/-4	1,264	384/44
Luang Prabang	Q_{1d}	14,510	15,637	1,127/8	13,848	-662/-5
	Q_{90d}	8,569	8,968	399/5	8,026	-543/-6
	Q_{1m}	907	751	-156/-17	1,183	276/30
	Q_{90m}	1,093	1,031	-62/-6	1,663	570/52
Vientiane	Q_{1d}	16,809	16,677	-133/-1	15,458	-1,352/-8
	Q_{90d}	10,577	10,396	-181/-2	8,668	-1,909/-18
	Q_{1m}	1,045	984	-61/-6	1,144	99/9
	Q_{90m}	1,230	1,265	35/3	1,508	278/23
Mukdahan	Q_{1d}	28,144	30,001	1,857/7	2,661	-1,483/-5
	Q_{90d}	19,685	20,824	1,139/6	18,247	-1,437/-7
	Q_{1m}	1,352	1,620	268/20	2,379	1,027/76
	Q_{90m}	1,619	1,905	286/18	2,917	1,298/80
Pakse	Q_{1d}	37,932	36,284	-1,649/-4	34,096	-3,836/-10
	Q_{90d}	25,218	25,764	546/2	22,384	-2,834/-11
	Q_{1m}	1,541	1,673	132/9	2,209	668/43
	Q_{90m}	1,846	1,659	-187/-10	2,851	1,005/54

Note: Q_{1d} and Q_{1m} were calculated using Equations (2–3), and Q_{90d} and Q_{90m} were calculated using Equations (4–6).

Supplementary Table S4. Change of water level, change of water level liked to discharge and channel deformation at $\mathbf{Q_{1d}}$, $\mathbf{Q_{90d}}$, $\mathbf{Q_{1m}}$ and $\mathbf{Q_{90m}}$ discharges during Period II and Period III at the five hydrological stations.

Hydrological Station	Change of water level	Change linked to discharge	Change linked to channel deformation	Change of water level	Change linked to discharge	Change linked to channel deformation	Change of water level	Change linked to discharge	Change linked to channel deformation	Change of water level	Change linked to discharge	Change linked to channel deformation
High-flow situation	Q_{1d} (Period II)			Q_{1d} (Period III)			Q_{90d} (Period II)			Q_{90d} (Period III)		
Chiang Saen	0.51	0.19	0.32	-2.25	-2.36	0.11	0.18	0.06	0.12	-1.44	-1.44	0.00
Luang Prabang	0.44	0.66	-0.22	-0.96	-0.93	-0.04	0.55	0.30	0.25	-1.04	-0.67	-0.37
Vientiane	0.26	-0.05	0.32	-0.55	-0.48	-0.07	0.30	-0.09	0.39	-0.83	-0.93	0.10
Mukdahan	0.10	0.51	-0.41	-1.22	-0.87	-0.35	0.11	0.36	-0.25	-1.20	-0.79	-0.41
Pakse	-0.31	-0.37	0.06	-0.15	-0.50	0.35	0.19	0.14	0.05	-0.71	-0.88	0.17
Low-flow situation	Q_{1m} (Period II)			Q_{1m} (Period III)			Q_{90m} (Period II)			Q_{90m} (Period III)		
Chiang Saen	-0.09	-0.32	0.23	0.55	0.49	0.06	0.12	-0.06	0.18	0.66	0.64	0.02
Luang Prabang	-0.18	-0.37	0.18	0.54	1.07	-0.53	0.14	-0.13	0.27	0.73	1.30	-0.57
Vientiane	0.01	-0.11	0.12	-0.02	0.31	-0.33	0.23	0.06	0.17	0.19	0.41	-0.22
Mukdahan	0.11	0.23	-0.13	0.28	0.61	-0.33	0.11	0.23	-0.12	0.41	0.76	-0.35
Pakse	0.21	0.09	0.12	0.15	0.34	-0.18	0.00	-0.12	0.12	0.55	0.72	-0.17

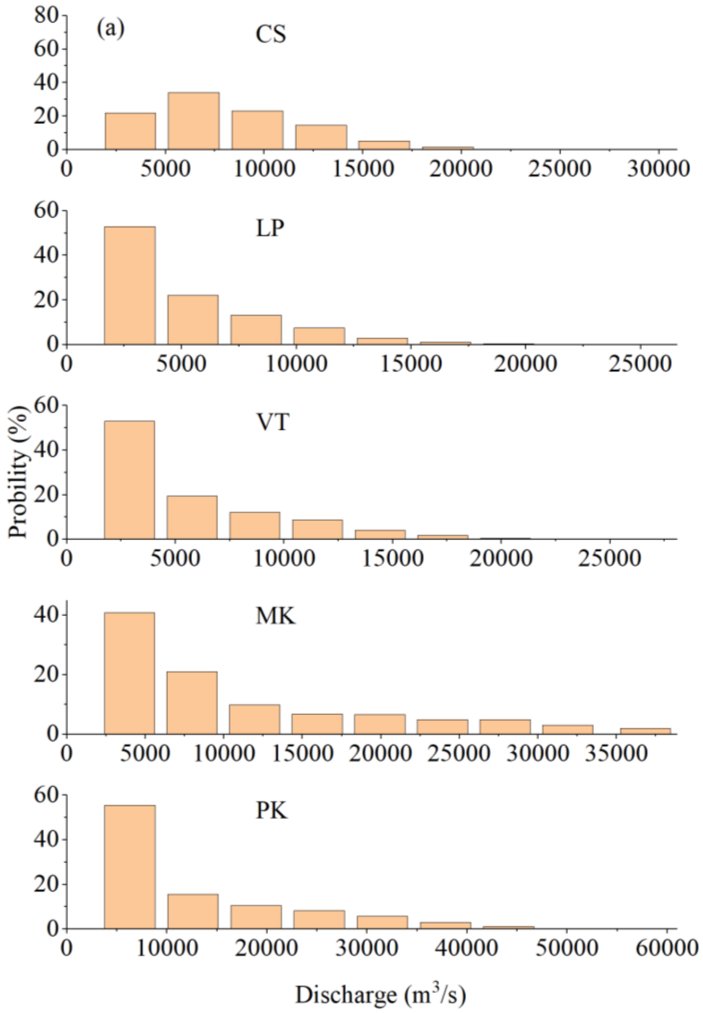

Supplementary Figure S1 Bar chart of discharge (Column a) and water level (Column b). Water level are data above the corresponding local datum.

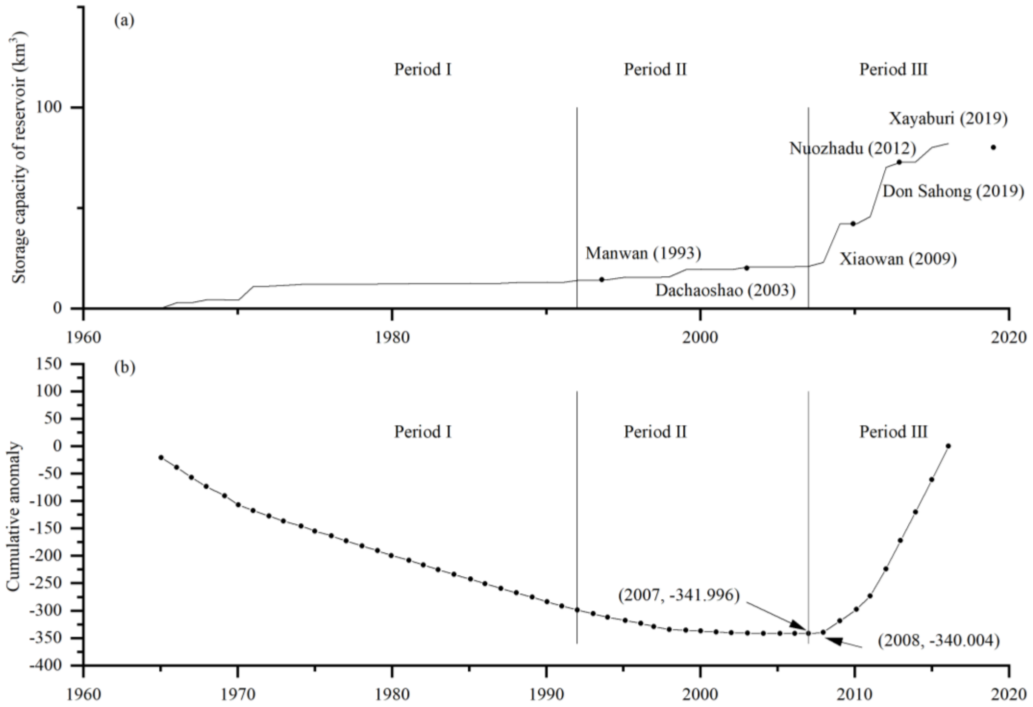

Supplementary Figure S2 Temporal variation of total storage capacity of reservoir (a) and anomalies of storage capacity of reservoir (b) by cumulative anomaly method. In plot (a), the number in parentheses is the year where the reservoir was commissioned. In plot (b), the numbers in parentheses are the year and the corresponding value by cumulative anomaly method.

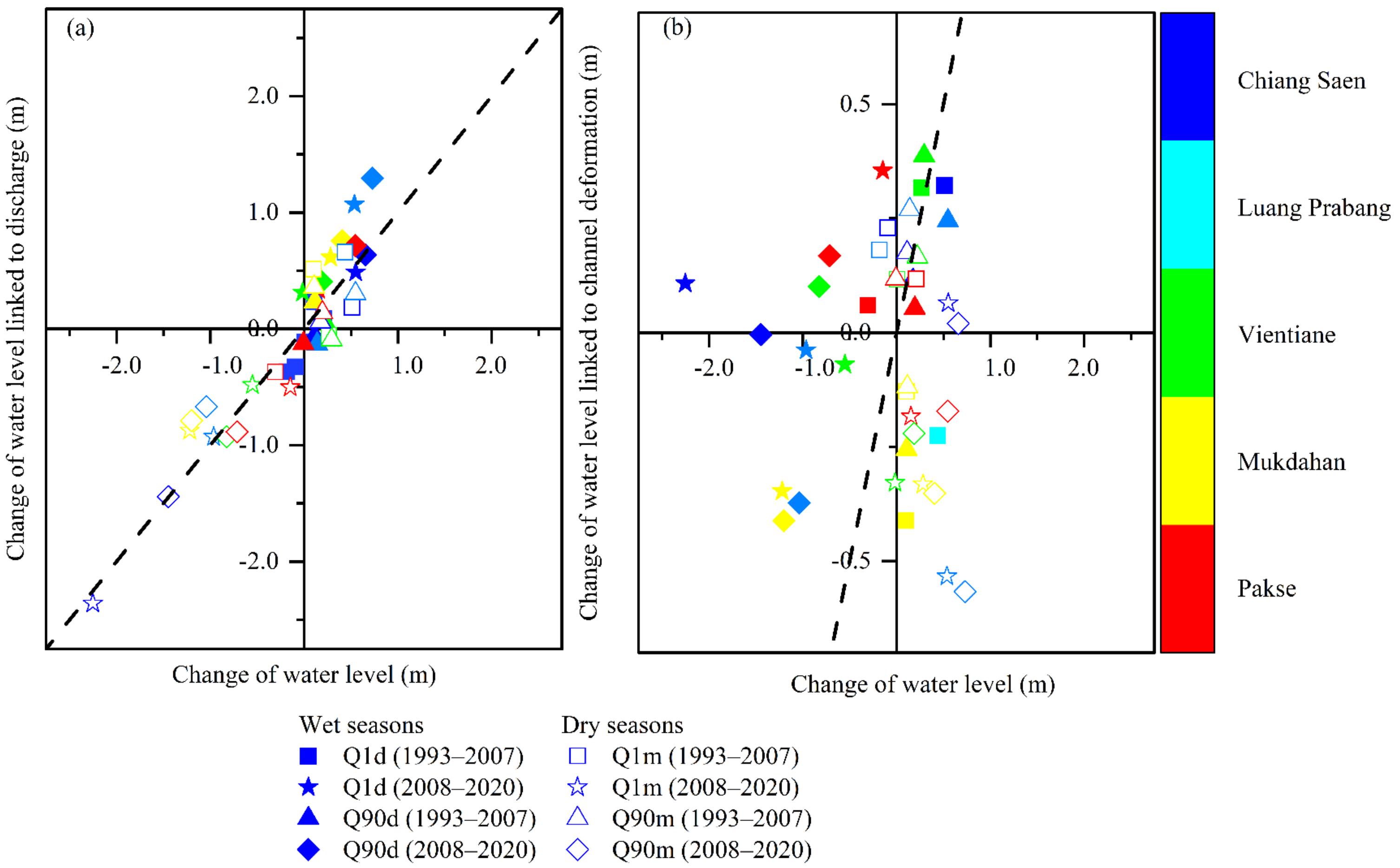

Supplementary Figure S3. Contributions of discharge and channel deformation to water level changes. Water level changes in $\mathbf{Q_{1d}}$, $\mathbf{Q_{90d}}$, $\mathbf{Q_{1m}}$ and $\mathbf{Q_{90m}}$ discharge at the five hydrological stations are shown. Different colors indicate different hydrological stations. Hollow and empty symbols represent wet and dry situation values, respectively. The dashed line indicates the 45 °line. Dots where both the x- and y-axis are positive or both negative values indicate that the effect of discharge or channel deformation is consistent with water level changes. In (a), the effect of

discharge is consistent with water level changes, indicating a dominant role of discharge in influencing water level change in both wet and dry situations. In (b), points with opposite values on the x- and y-axis indicates a modification of change of water level by channel deformation. The proportion of these points is 60% and 40% in dry and wet situations, respectively.

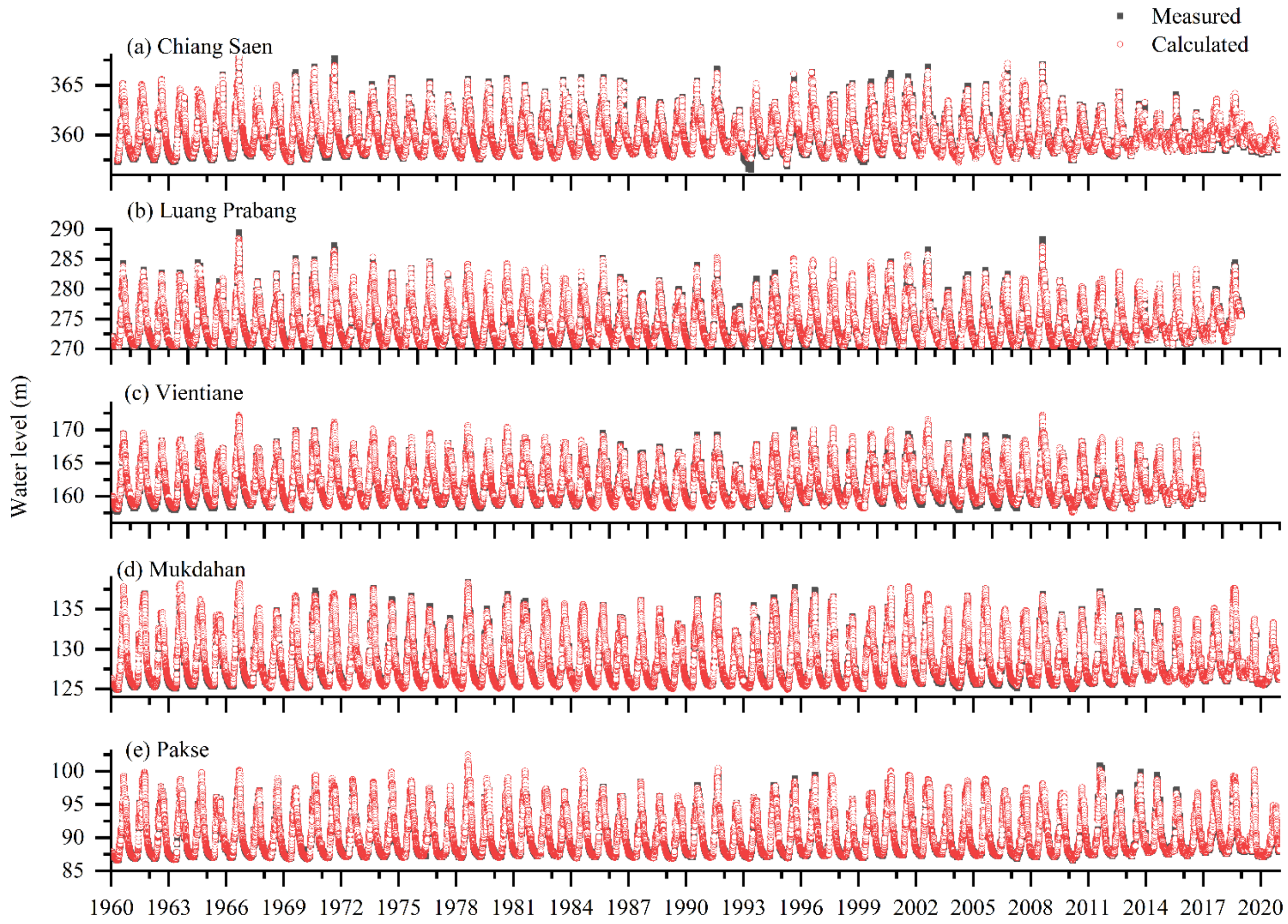

Supplementary Figure S4. Evaluation of fitted rating curves during Period I, Period II, and Period III at the five hydrological stations. The difference between calculated water levels and observed values during the flood situation ranged from -0.09~0.14 m (-0.03%~0.15%), while the difference during the dry situation ranged from -0.03~0.06 m (0.07%~0.04%).

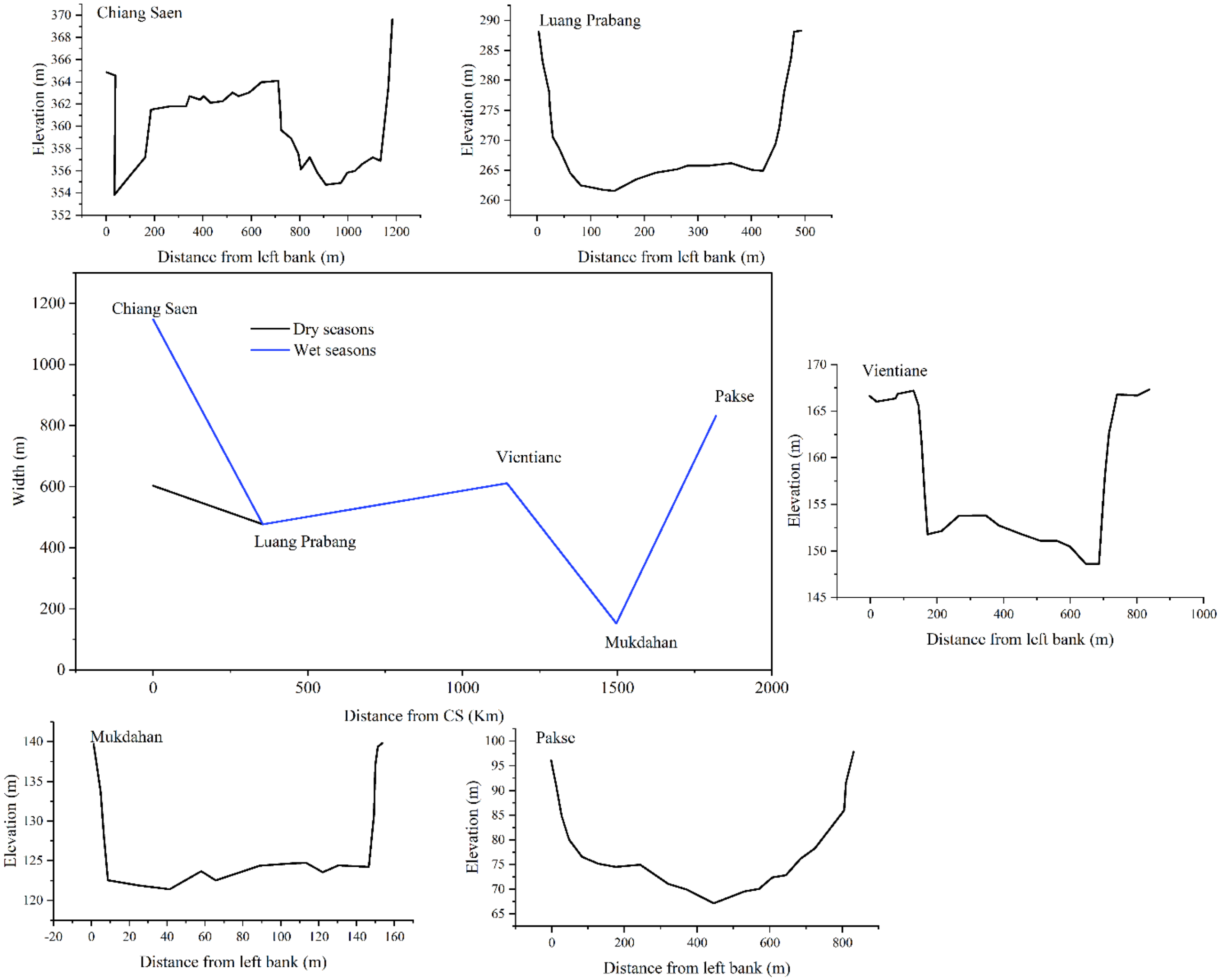

Chiang Saen
Elevation (m)
Distance from left bank (m)
Luang Prabang
Elevation (m)
Distance from left bank (m)
Chiang Saen
Dry seasons
Wet seasons
Pakse
Vientiane
Luang Prabang
Mukdahan
Width (m)
Distance from CS (Km)
Vientiane
Elevation (m)
Distance from left bank (m)
Mukdahan
Elevation (m)
Distance from left bank (m)
Pakse
Elevation (m)
Distance from left bank (m)

Supplementary Figure S5. Width and channel cross sections of the five hydrology stations along the LM River. Data are drawn from Hou[2]. As its bifurcation channel, the cross-sectional width at Chiang Saen changes may change dramatically when discharge is smaller than bankfull discharge and water level is lower than bankfull elevation.

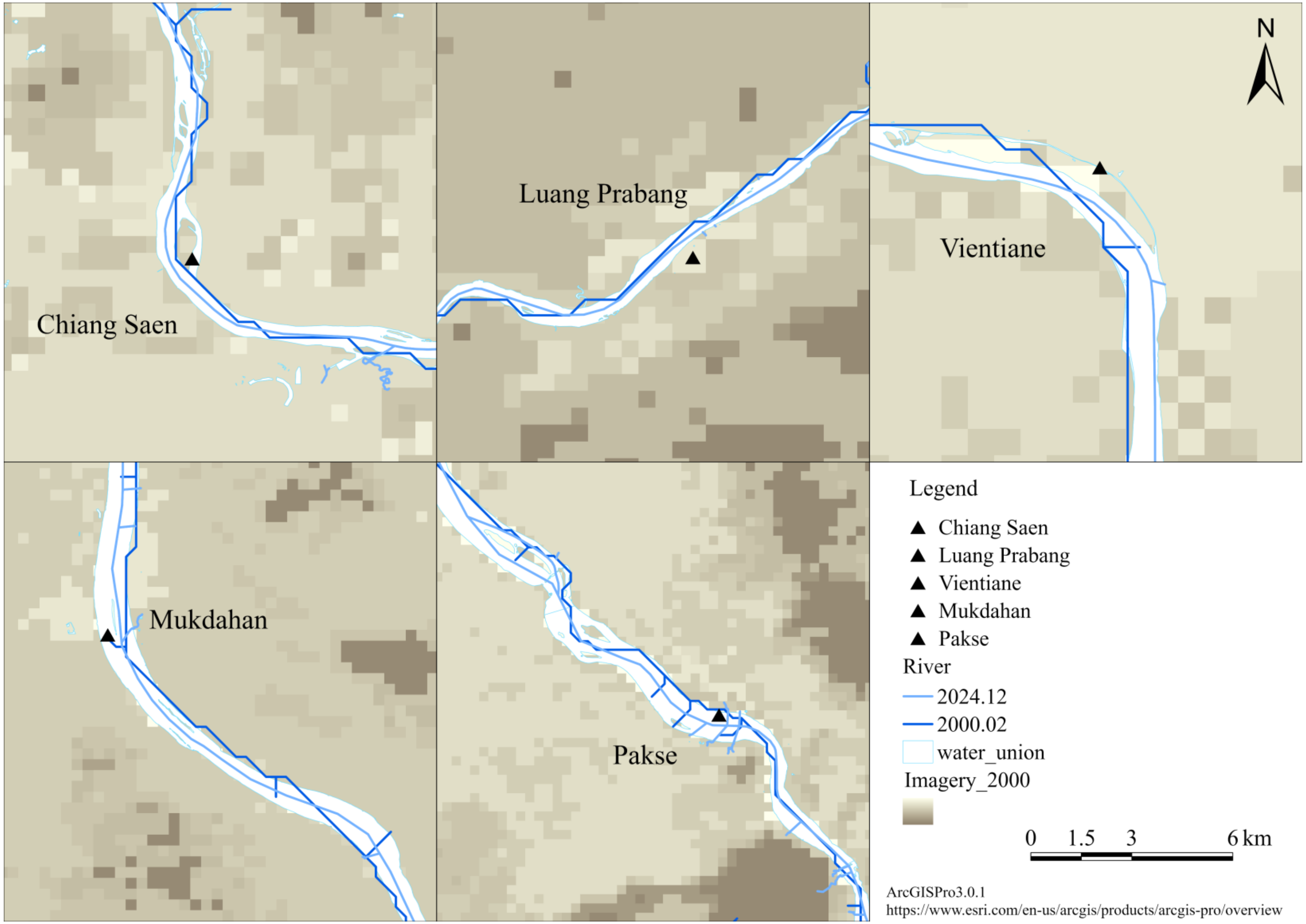

ArcGISPro3.0.1
https://www.esri.com/en-us/arcgis/products/arcgis-pro/overview

Supplementary Figure S6 Planform variation of main flow around the five hydrology stations. Images are drawn from 2024 and 2000, respectively. Flow route in 2024 and 2000 are topological river vector data in December 2024 (https://openstreetmap.org) and February 2000 (https://www.hydrosheds.org/productes/hydrotivers) respectively. Water_union is the topological river vector data in 2024 from https://openstreetmap.org. Imagery_2000 is ESA-Sentinel-2-raster imagery data from https://dataspace.copernicus.eu/. ArcGISPro3.0.1 (https://www.esri.com/en-us/arcgis/product/arcgis-pro/overview) was adopted to create this figure.

Reference

1 Chua, S. D. X. & Lu, X. X. What can stage curves tell us about water level changes? Case study of the Lower Mekong Basin. *Catena* **216**, 106385 (2022).
2 Hou, S.Y. Research on characteristics of water and sediment and their responses to environment changes in Lancang-Mekong River basin. PhD Thesis, Tsinghua University (2021).
